# Evaluation of the performance of Human Papillomavirus testing in paired urine and clinician-collected cervical samples among women aged over 30 years in Bhutan

**DOI:** 10.1186/s12985-017-0744-2

**Published:** 2017-04-08

**Authors:** Ugyen Tshomo, Silvia Franceschi, Tshokey Tshokey, Tashi Tobgay, Iacopo Baussano, Vanessa Tenet, Peter J. F. Snijders, Tarik Gheit, Massimo Tommasino, Alex Vorsters, Gary M. Clifford

**Affiliations:** 1 Department of Obstetrics & Gynaecology, Jigme Dorji Wangchuck National Referral Hospital, Thimphu, Bhutan; 2 International Agency for Research on Cancer, 150 cours Albert Thomas, 69372 Lyon cedex 08, France; 3 Department of Pathology & Laboratory Medicine, Jigme Dorji Wangchuck National Referral Hospital, Thimphu, Bhutan; 4 Department of Pathology, VU University Medical Center, De Boelelaan 1117, 1081 HV Amsterdam, The Netherlands; 5 Centre for the Evaluation of Vaccination, Vaccine and Infectious Disease Institute, University of Antwerp, Antwerp, Belgium

**Keywords:** Human papillomavirus, Urine, Cervical cancer, Bhutan

## Abstract

**Background:**

Urine sampling may offer a less invasive solution than cervical sampling to test for human papillomavirus (HPV) for HPV vaccine impact monitoring.

**Methods:**

Paired samples of urine and exfoliated cervical cells were obtained for 89 women with history of high-risk (HR) HPV-positive normal cytology in Bhutan. Urine sampling protocol included self-collection of first-void urine immediately into a conservation medium and procedures to optimize DNA yield. Colposcopical abnormalities were biopsied. Two HPV assays were used: a multiplex type-specific PCR (E7-MPG) and a less analytically sensitive GP5+/6+ PCR followed by reverse line blot.

**Results:**

HPV positivity for 21 types common to both assays was similar in urine and cells by E7-MPG (62.9% and 57.3%, respectively, *p* = 0.32) but lower in urine by GP5+/6+ (30.3% and 40.4%, *p* = 0.05). HPV6/11/16/18 positivity did not significantly differ between urine and cells by either assay. Sensitivity of urine (using cells as gold standard) to detect 21 HPV types was 80% and 58% for E7-MPG and GP5+/6+, respectively, with specificity 61% and 89%. HPV type distribution in urine and cells was similar, regardless of assay. The 5 detected CIN3+ were HR-HPV positive in cells by both assays, compared to 4 and 3 by E7-MPG and GP5+/6+, respectively, in urine samples.

**Conclusion:**

For the monitoring of vaccine impact, we demonstrate validity of a urine sampling protocol to obtain HPV prevalence data that are broadly comparable to that from cervical cells. However, detection of HPV in urine varies according to assay sensitivity, presumably because low level infections are frequent.

## Background

Confidence in the use of urine samples for the detection of human papillomavirus (HPV) has been increasing in recent years. Systematic reviews have shown a reasonable concordance with cervical cells for HPV positivity in women [1–3], with some important steps to improve sensitivity including use of first-void rather than random or mid-stream samples [1, 4, 5], and avoidance of DNA degradation by the immediate use of a conservation medium [6].

Using a device for self-collection of first-void urine and implementation of optimized procedures for urine sample management, we have recently reported good acceptability and performance of a protocol of HPV testing from urine to monitor HPV vaccine impact in young women in Bhutan and Rwanda [7]. Indeed, urine sampling offers a less invasive solution than cervical sampling to obtaining information from a representative sample of young women reluctant to accept a gynaecological examination. Other urine testing protocols are being used to monitor HPV vaccine impact in females [8, 9] and males [10] in high-income settings, and are also being evaluated as alternatives to cervical sampling for cervical cancer screening [5, 11–13].

However, the choice of assay for HPV testing in urine can impact estimates of epidemiological associations and HPV vaccine effectiveness [7], so that the relative merits and dangers of high analytical sensitivity remains unclear for monitoring the impact of HPV vaccine, and have been little studied in cervical cancer screening based on urine sampling.

We aimed to estimate the performance of the above mentioned urine sampling protocol directly against paired cervical cytological samples, and using two HPV assays of differing sensitivity, namely GP5+/6+RLB, an assay developed for clinical specificity in HPV-based cervical screening and E7-MPG, which has greater analytical sensitivity [14]. This was done in women undergoing colposcopy, with histological confirmation of lesions, in a cohort of well-characterised women in Bhutan. Our hypothesis was that our protocol for urine sampling should give equivalent HPV results to the cervix.

## Methods

### Population

During a population-based study in 2012, 2505 women aged 18–69 years were invited for the collection of exfoliated cervical cells for a PAP smear and HPV testing by GP5+/6+RLB [15]. Women with abnormal cytology were immediately recalled for colposcopy and, if necessary, treatment. When HPV results became available, approximately 2 years after the original sample collection, all high-risk (HR)-HPV-positive women aged over 30 with normal cytology were also recalled. Of 115 HR-HPV-positive women with normal cytology, 95 women attended a colposcopy examination at Jigme Dorji Wangchuck National Referral Hospital (JDWNRH), Thimphu, between May and August 2014, at which time a repeat cervical cytology and urine sample were obtained (see below). All women with abnormal colposcopical findings underwent biopsy and appropriate treatment by local gynaecologists. Histological confirmation of cervical tissue was performed at JDWNRH. All participants signed informed consent forms and the study had the approval of both the Research Ethical Board of the Bhutan Ministry of Health and the IARC Ethics Committee.

### Urine collection and DNA extraction

Immediately prior to colposcopy, participants self-collected a urine sample using a device (Colli Pee™, Novosanis) designed to collect the first 14 ml of first-void urine immediately into 7 ml of a urine-conservation medium to avoid DNA degradation [7]. DNA extraction was performed at the Centre for the Evaluation of Vaccination, University of Antwerp, Belgium, as described elsewhere [7]. Briefly, in order to concentrate all DNA, including cell free DNA fragments, 4 ml of urine sample was centrifuged at 4000 g for 20 mins in an Amicon Ultra-4 50 K filter device. Centrifugation was repeated for 10 min if remaining volume on the filter was more than 1 ml. After filtration, 2 ml of NucliSENS Lysis Buffer was added to the concentrate retained on the filter and incubated for 10 min at room temperature. All material was subsequently transferred to NucliSENS Lysis Buffer and DNA extraction was performed using the generic easyMAG off-board lysis protocol. DNA was subsequently eluted in 55 μl of elution buffer.

### Collection of cervical cells during colposcopy

After the urine collection, during colposcopy, a cytobrush (Cervex-Brush, Rovers Medical Devices, The Netherlands) was used for the collection of exfoliated cervical cells. After preparation of a conventional Pap smear, the brush containing cellular material was placed in a vial containing 20 ml PreservCyt medium. DNA was extracted from the PreservCyt sample in the Department of Pathology at the VU University Medical Centre, Amsterdam, using magnetic beads (Macherey Nagel) on a robotic system (Hamilton Star).

### HPV testing and genotyping

Two methods of different analytical sensitivity were used for HPV DNA testing.

A type specific E7 PCR bead-based multiplex genotyping assay (E7-MPG) was performed at IARC, Lyon using a Luminex bead-based platform [14]. The assay detects DNA from 21 HPV types (6, 11, 16, 18, 26, 31, 33, 35, 39, 45, 51, 52, 53, 56, 58, 59, 66, 68, 70, 73 and 82). Two β-globin primers are included to control DNA quality. This assay is known to be more sensitive than GP5+/6+ in detecting low viral copy numbers and, in particular, in detecting individual HPV types in multiple-type infections [14].

In the Department of Pathology at the VU University Medical Centre, Amsterdam, β-globin polymerase chain reaction (PCR) analysis was conducted to confirm the presence of human DNA in all specimens [16] and a general primer GP5+/6+ -mediated PCR was used to amplify HPV DNA. HPV positivity was assessed by hybridization of PCR products in an enzyme immunoassay with two oligoprobe cocktails that, together, detect 44 mucosal HPV types. Subsequent HPV genotyping was conducted by reverse-line blot hybridization of GP5+/6+ PCR products as described previously [14].

### Statistical analyses

HPV-positivity refers only to positivity for the 21 HPV types in common to both HPV genotyping methods (6, 11, 16, 18, 26, 31, 33, 35, 39, 45, 51, 52, 53, 56, 58, 59, 66, 68, 70, 73 and 82). Other HPV types detected by GP5+/6+RLB were ignored.

Type-specific positivity is compared between urine and cells by urine:cells prevalence ratios (PRs), as well as graphically. *P*-values from McNemar’s test for paired nominal data was used to evaluate the homogeneity of HPV findings between urine and cells by each assay. Graphs include two lines: a dotted line represents a theoretical scenario where types are detected equally in both samples, and that corresponds to a urine:cell PR of one; a solid line represents the slope of a linear regression passing through the origin and the 21 type-specific points, hence representing the average urine:cell PR. The strength of the association of this linear regression was assessed by using the coefficient of determination R^2^ or explained variation. Finally, sensitivity and specificity was computed for HPV detection in urine compared to cervical cells, i.e., the gold standard in cervical HPV-based screening.

## Results

Of 95 HR-HPV-positive women with normal cytology recalled for colposcopy, six had β-globin-negative urine samples invalid for HPV testing, leaving 89 women with paired urine and cervical samples. Median age was 39 years (5–95 percentile = 30–54 years), 83% reported only 1 lifetime sexual partner, and none were vaccinated against HPV.

HPV positivity for 21 types common to both assays is shown in Table 1, and was similar in urine (62.9%) and cells (57.3%) by the E7-MPG assay (urine:cell PR = 1.10, *p* = 0.32), whereas it was lower in urine (30.3%) than in cells (40.4%) by the GP5+/6+ test (PR = 0.75, *p* = 0.05). HPV positivity for vaccine types 6/11/16/18 was similar in urine and cells by both E7-MPG (25.8% and 23.6%, respectively, PR = 1.09, *p* = 0.48), and by GP5+/6+ (15.7% and 21.3%, PR = 0.74, *p* = 0.14) (Table 1). The prevalence of multiple HPV infection was higher for E7-MPG (22.5% and 19.1% in urine and cells, respectively), than for GP5+/6+ (6.7% and 7.9%, respectively). Table 1 also shows sensitivity and specificity of HPV detection in urine, versus cervical cell samples as a gold standard, by assay. The sensitivity for all the 21 common types was higher for E7-MPG (80%) than GP5+/6+ (58%) whereas specificity was lower (61% versus 89%). Restricting the analysis to HPV6/11/16/18, the difference in sensitivity was confirmed (86% by E7-MPG versus 58% by GP5+/6+) whereas specificity was more similar (93% and 96%, respectively).

**Table 1:** Comparison of agreement for HPV detection in urine versus cells, for E7-MPG and GP5+/6+ tests

		HPV result (n) urine/cells				Urine positivity	Cells positivity	Urine:cells Prevalence ratio	McNemar *p*-value	HPV detection in urine, versus cells as gold standard	
		+/+	-/-	+/-	-/+					Sensitivity	Specificity
21 HPV types	E7-MPG	41	23	15	10	62.9%	57.3%	1.10	0.32	80 (67–90)	61 (43–76)
	GP5+/6+RLB	21	47	6	15	30.3%	40.4%	0.75	0.05	58 (41–75)	89 (77–96)
						2.08*	1.42*				
HPV6/11/16/18	E7-MPG	18	63	5	3	25.8%	23.6%	1.09	0.48	86 (64–97)	93 (84–98)
	GP5+/6+RLB	11	67	3	8	15.7%	21.3%	0.74	0.14	58 (33–80)	96 (88–99)
						1.64*	1.11*				

*HPV* human papillomavirus *E7-MPG:GP5+/6+RLB ratio

**Fig. 1:**
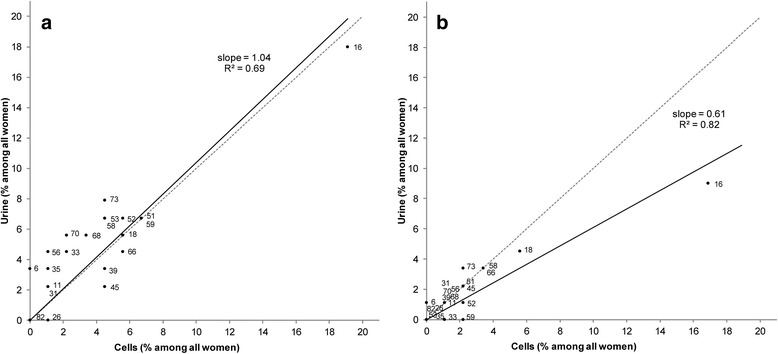
HPV type-specific positivity compared among 89 paired urine and cervical samples^a^, **a** E7-MPG, **b** GP5+/6+. ^a^Dotted lines represent a theoretical scenario where types are detected equally in both samples (urine:cell PR = 1); solid lines represent the slope of a linear regression passing through the origin and the 21 type-specific points (average urine:cell PR)

Figure 1 compares HPV type-specific positivity in urine versus cells, separately for E7-MPG and GP5+/6+ assays. For E7-MPG, the 21 HPV-types were found, on average, as frequently in urine as in cells (i.e. the slope of the linear regression line is close to one (Fig. 1a). For GP5+/6+, however, HPV types tended to be detected less frequently in urine than in cells and the average urine:cell PR across the 21 types (i.e. the slope of the linear regression line) was 0.61 (Fig. 1b). HPV16 was by far the most commonly detected HPV type, irrespective of the HPV assay or type of sample. There was no evidence for any HPV type to be differentially detected in urine or cells by either assay as shown by the relatively high R^2^ values (0.69 for E7-MPG and 0.82 for GP5+/6+).

Eleven cervical intraepithelial neoplasia (CIN) grade 1, 6 CIN2, 4 CIN3 and 1 invasive cancer were histologically diagnosed among these 89 patients. Table 2 shows the detection of HPV types in the 5 CIN3 or worse (+) cases. All 5 cell samples were HR-HPV-positive, both for E7-MPG and GP5+/6+. For urine samples, 4 out of 5 CIN3+ were HR-HPV-positive by E7-MPG, and 3 out of 5 were HR-HPV-positive by GP5+/6+. Of note, HPV positive urine and cell samples were always positive for the same type that was found in the normal cytology sample collected, on average, 2 years earlier.

**Table 2:** Description of HPV types found in women with histologically confirmed CIN3 or worse at colposcopy

HPV result in normal cytology (~2 years prior)	Histological diagnosis	E7-MPG		GP5+/6+RLB	
		urine	cells	urine	cells
18,56	CIN3	56	56	56	56
16	CIN3	16,35,59	16,59	16	16
18	CIN3	16,18	18,26,45	18	18
45	CIN3	negative	33,45,51,53,66,73	negative	45, 66
51	Cancer	51,66,70	51,66	negative	51

*CIN* cervical intraepithelial neoplasia; *HPV* human papillomavirus; *HR* high-risk

Underline = type found in HR-HPV-positive normal cytology sample taken ~2 years earlier

## Discussion

We confirmed a >70% concordance of HPV testing between urine and paired cervical cell samples among women in Bhutan using two differently sensitive assays. Furthermore, whereas previous studies have almost all reported a moderate under detection of HPV in urine compared to paired cervical cell samples [1], we report that the combination of a strict protocol for urine sampling and a highly sensitive HPV detection assay (E7-MPG), resulted in detection of more HPV in urine than in cervical cells. This finding matches that of a recent study in Colombia using a similar urine sampling procedure and HPV assay (PR = 1.08 for any HPV) [17]. Neither in the present, nor the Colombian study, was there any clear evidence that HPV type distribution varied between the two sample types, suggesting that urine is broadly representative of the types collected at the cervix, at least with respect to the 21 types evaluated by both E7-MPG and GP5+/6+. Improvements in HPV detection from urine over previous studies are expected partly to be due to optimized urine sampling procedures. These include: 1) collection of first-void, rather than random or mid-stream, urine [1, 4, 5]; 2) avoidance of DNA degradation through the use of urine-conservation medium and buffer in both urine collection and processing [6]; 3) sufficient volume of urine to allow subsequent sample concentration [6]; and 4) recovery of cell-free HPV DNA in addition to cell-associated DNA [6].

However, urine:cell PR can also vary according to the HPV assay used. When these same samples were tested by GP5+/6+, an assay developed for clinical specificity in HPV-based cervical screening, HPV prevalence was lower than by E7-MPG, as expected. However, the difference in HPV prevalence between the two tests was greater in urine than in cells. This resulted in significantly lower detection of HPV in urine compared to cervical cells when relying on GP5+/6+, which is similar to findings of a meta-analysis of previous studies [1], although comparisons are difficult due to variations in urine sampling and HPV testing protocols used.

We have recently shown that E7-MPG infections non-detected by GP5+/6+RLB are associated with low viral copy number (as measured by median MFI values) [14], a finding that has been reported previously [18]. So the large fraction of E7-MPG positive urine samples that are negative by GP5+/6+ in the present study indirectly confirms that HPV DNA detected in urine often reflects the presence of low viral copy numbers. Indeed, we have previously shown that estimates of vaccine effectiveness against HPV6/11/16/18 using the same urine sampling procedure were lower using the more sensitive E7-MPG than GP5+/6+, and speculated that it was due to increased detection of low-level HPV infections against which vaccine efficacy is unclear [7].

This study was neither designed nor powered to evaluate the utility of urine testing for detection of precancerous lesions in cervical cancer screening. Indeed, to obtain more information of the performance of cells and urine samples, we chose a group of women especially likely to be HPV-positive, and numbers were small. Nevertheless, we notice anecdotally that all 5 cervical cell samples from CIN3+ cases were HR-HPV-positive, irrespective of the test used and, of these, 4 out of 5 were positive by E7-MPG and 3 out of 5 by GP5+/6+RLB when tested from urine. Some previous studies have shown similar results of CIN3 sensitivity (74–100%) [11, 19, 20], whereas the largest study to date, albeit that did not use any DNA preservation medium, reported CIN3+ sensitivity of 51% [12].

## Conclusion

In conclusion, we demonstrate the validity of a protocol of urine self-collection and HPV testing that we are already using for the long-term monitoring of vaccine impact in Bhutan and Rwanda [7]. In young women that are unwilling to undergo a gynaecological examination, this urine testing protocol should give results that are broadly representative of HPV at the cervix, but findings can be expected to be affected by HPV assay sensitivity.

## Abbreviations

CIN: Cervical intraepithelial neoplasia
HPV: Human papillomavirus
HR: High-risk
JDWNRH: Jigme Dorji Wangchuck National Referral Hospital
LMIC: Low- and middle-income country

## References

[1] Pathak N, Dodds J, Zamora J, Khan K (2014). Accuracy of urinary human papillomavirus testing for presence of cervical HPV: systematic review and meta-analysis. BMJ.

[2] Enerly E, Olofsson C, Nygard M (2013). Monitoring human papillomavirus prevalence in urine samples: a review. Clin Epidemiol.

[3] Vorsters A, Micalessi I, Bilcke J, Ieven M, Bogers J, Van Damme P (2012). Detection of human papillomavirus DNA in urine. A review of the literature. Eur J Clin Microbiol Infect Dis.

[4] Vorsters A, Van Damme P, Clifford G (2014). Urine testing for HPV: rationale for using first void. BMJ.

[5] Senkomago V, Des Marais AC, Rahangdale L, Vibat CR, Erlander MG, Smith JS (2016). Comparison of urine specimen collection times and testing fractions for the detection of high-risk human papillomavirus and high-grade cervical precancer. J Clin Virol.

[6] Vorsters A, Van den Bergh J, Micalessi I, Biesmans S, Bogers J, Hens A, De Coster I, Ieven M, Van Damme P (2014). Optimization of HPV DNA detection in urine by improving collection, storage, and extraction. Eur J Clin Microbiol Infect Dis.

[7] Franceschi S, Chantal Umulisa M, Tshomo U, Gheit T, Baussano I, Tenet V, Tshokey T, Gatera M, Ngabo F, Van Damme P (2016). Urine testing to monitor the impact of HPV vaccination in Bhutan and Rwanda. Int J Cancer.

[8] Kavanagh K, Sinka K, Cuschieri K, Love J, Potts A, Pollock KG, Cubie H, Donaghy M, Robertson C (2013). Estimation of HPV prevalence in young women in Scotland; monitoring of future vaccine impact. BMC Infect Dis.

[9] Sonnenberg P, Clifton S, Beddows S, Field N, Soldan K, Tanton C, Mercer CH, da Silva FC, Alexander S, Copas AJ (2013). Prevalence, risk factors, and uptake of interventions for sexually transmitted infections in Britain: findings from the National Surveys of Sexual Attitudes and Lifestyles (Natsal). Lancet.

[10] Chow EP, Machalek DA, Tabrizi SN, Danielewski JA, Fehler G, Bradshaw CS, Garland SM, Chen MY, Fairley CK. Quadrivalent vaccine-targeted human papillomavirus genotypes in heterosexual men after the Australian female human papillomavirus vaccination programme: a retrospective observational study. Lancet Infect Dis. 2017;17:68–77. 10.1016/S1473-3099(16)30116-5 27282422

[11] Sahasrabuddhe VV, Gravitt PE, Dunn ST, Robbins D, Brown D, Allen RA, Eby YJ, Smith KM, Zuna RE, Zhang RR (2014). Evaluation of clinical performance of a novel urine-based HPV detection assay among women attending a colposcopy clinic. J Clin Virol.

[12] Stanczuk G, Baxter G, Currie H, Lawrence J, Cuschieri K, Wilson A, Arbyn M (2016). Clinical validation of hrHPV testing on vaginal and urine self-samples in primary cervical screening (cross-sectional results from the Papillomavirus Dumfries and Galloway-PaVDaG study). BMJ Open.

[13] Ducancelle A, Reiser J, Pivert A, Le Guillou-Guillemette H, Le Duc-Banaszuk AS, Lunel-Fabiani F (2015). Home-based urinary HPV DNA testing in women who do not attend cervical cancer screening clinics. J Infect.

[14] Clifford GM, Vaccarella S, Franceschi S, Tenet V, Umulisa MC, Tshomo U, Dondog B, Vorsters A, Tommasino M, Heideman DA (2016). Comparison of two widely used human papillomavirus detection and genotyping methods, GP5+/6 + -based PCR followed by reverse line blot hybridization and multiplex type-specific E7-based PCR. J Clin Microbiol.

[15] Tshomo U, Franceschi S, Dorji D, Baussano I, Tenet V, Snijders PJ, Meijer CJ, Bleeker MC, Gheit T, Tommasino M, Clifford GM (2014). Human papillomavirus infection in Bhutan at the moment of implementation of a national HPV vaccination programme. BMC Infect Dis.

[16] de Roda Husman AM, Snijders PJ, Stel HV, van den Brule AJ, Meijer CJ, Walboomers JM (1995). Processing of long-stored archival cervical smears for human papillomavirus detection by the polymerase chain reaction. Br J Cancer.

[17] Combita AL, Gheit T, Gonzalez P, Puerto D, Murillo RH, Montoya L, Vorsters A, Van Keer S, Van Damme P, Tommasino M (2016). Comparison between urine and cervical samples for HPV DNA detection and typing in young women in Colombia. Cancer Prev Res (Phila).

[18] Bissett SL, Howell-Jones R, Swift C, De SN, Biscornet L, Parry JV, Saunders NA, Nathan M, Soldan K, Szarewski A (2011). Human papillomavirus genotype detection and viral load in paired genital and urine samples from both females and males. J Med Virol.

[19] Piyathilake CJ, Badiga S, Chambers MM, Brill IK, Matthews R, Partridge EE (2016). Accuracy of urinary human papillomavirus testing for the presence of cervical human papillomaviruses and higher grades of cervical intraepithelial neoplasia. Cancer.

[20] Nicolau P, Mancebo G, Agramunt S, Sole-Sedeno JM, Bellosillo B, Muset MM, Lloveras B, Alameda F, Carreras R (2014). Urine human papillomavirus prevalence in women with high-grade cervical lesions. Eur J Obstet Gynecol Reprod Biol.

